# Fighting for independence

**DOI:** 10.1186/s12915-016-0227-8

**Published:** 2016-01-19

**Authors:** Emma Saxon

**Affiliations:** BMC Biology, BioMed Central, 236 Gray’s Inn Road, London, WC1X 8HB UK

## Abstract

Male crickets (*Gryllus bimaculatus*) establish dominance hierarchies within a population by fighting with one another. Larger males win fights more frequently than their smaller counterparts, and a previous study found that males recognise one another primarily through sensory input from the antennae. This study therefore investigated whether the success of larger crickets is influenced by sensory input from the antennae, in part by assessing the number of fights that large ‘antennectomized’ crickets won against small crickets, compared with the number that large, intact crickets won. The success rate was significantly lower in antennectomized males, though they still won the majority of fights (73/100 versus 58/100, Fisher’s exact test *P* < 0.05); the authors thus conclude that sensory input from the antennae affects the fighting success of large males, but that other size-related factors also play a part.

## Comment

**Fig. 1:**
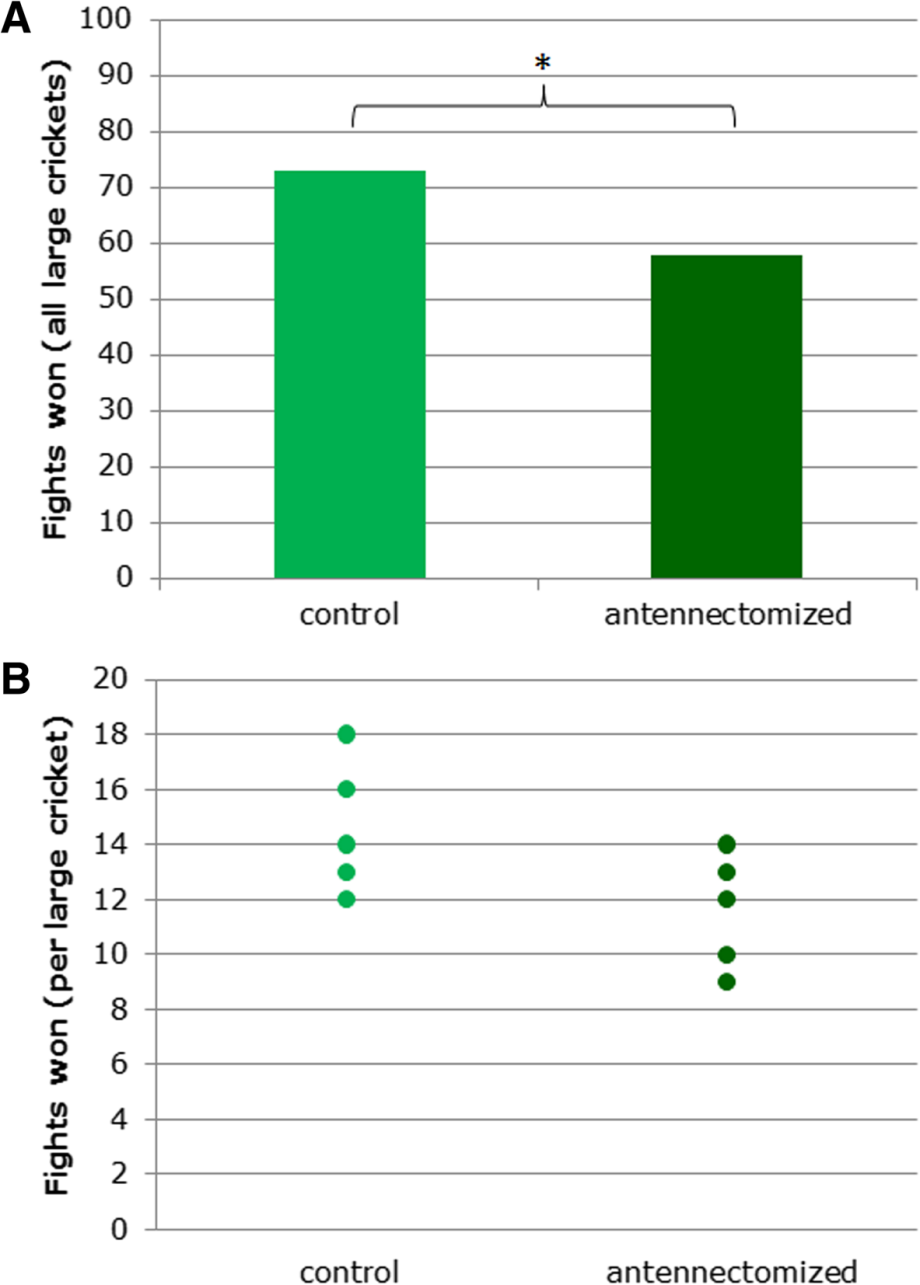
**a** The total number of fights won by large crickets against small crickets in antennectomized versus control, sham-operated groups. **b** The number of fights won by each large cricket in antennectomized versus control groups. Fisher’s exact test *P < 0.05

The outcome of a study can be misleading if the measurements it is based on are not independent — in other words, if they are related to or affected by one another. Here, the authors tested the fighting ability of large males (in the top 20^th^ percentile for weight) against their smaller counterparts. Large and small crickets from a sample population of 50 were randomly assigned to one of two groups, each containing 5 large and 20 small crickets. In one group, the antennae of the large crickets were removed, while in the other, the large crickets received a sham operation as a control. The authors recorded the outcome of every possible fight between large and small crickets in the antennectomized and control groups (totalling 5 × 20 = 100 fights per group), and found that the large control crickets won more frequently than the large crickets lacking antennae, as shown in Fig. 1a (73/100 versus 58/100 wins, Fisher’s exact test *P* < 0.05).

Fisher’s exact test assumes that all observations are independent, but this was not the case for the fight outcome data: each large cricket took part in 20 fights, the results of which were thus influenced by the fighting ability of a single large cricket. This is stated in the methods section, but cannot be inferred from Fig. 1a. Had the authors plotted the proportion of fights won by each cricket, shown in Fig. 1b, it would have been clear that the independent samples in this study were crickets rather than fights. The authors should have used an alternative statistical test such as Student’s t-test (which returns a non-significant result, *P* = 0.068); or, in order to use Fisher’s exact test appropriately, they could instead have recorded the outcomes from single pairs of crickets, with no cricket fighting more than once. In that case, more crickets should be tested for the data to be reliable.

The problem of non-independence is frequent in ecological or behavioural studies: these authors might also have compared wins and losses between large and small crickets pitted directly against one another. A win for a large cricket would entail a loss for the small cricket; thus, these outcomes would also be dependent on one another. More complex cases of non-independence can be more difficult to identify, and thus overlooked — studies modelling behaviour in animals that live in groups, for example, can rarely assume that individuals are independent, as they are often positively or negatively influenced by other group members. For example, group members might feed together to avoid predation, or conversely allow more dominant individuals to take priority over shared resources to avoid aggressive competition. These potential confounding factors should be accounted for in the study design, before any data are collected and analysed.

